# Specificity in genetic and environmental risk for prescription opioid misuse and heroin use

**DOI:** 10.1017/S003329172300034X

**Published:** 2023-03-22

**Authors:** Genevieve F. Dash, Ian R. Gizer, Nicholas G. Martin, Wendy S. Slutske

**Affiliations:** 1 Department of Psychological Sciences, University of Missouri, Columbia, MO 65211, USA;; 2 QIMR Berghofer, Brisbane, Queensland 4006, Australia; 3 Department of Family Medicine and Community Health and Center for Tobacco Research and Intervention, University of Wisconsin, Madison, WI 53711, USA

**Keywords:** Drug use, heroin, multivariate, prescription opioids, twin study

## Abstract

**Background.:**

Many studies aggregate prescription opioid misuse (POM) and heroin use into a single phenotype, but emerging evidence suggests that their genetic and environmental influences may be partially distinct.

**Methods.:**

In total, 7164 individual twins (84.12% complete pairs; 59.81% female; mean age = 30.58 years) from the Australian Twin Registry reported their lifetime misuse of prescription opioids, stimulants, and sedatives, and lifetime use of heroin, cannabis, cocaine/crack, illicit stimulants, hallucinogens, inhalants, solvents, and dissociatives via telephone interview. Independent pathway models (IPMs) and common pathway models (CPMs) partitioned the variance of drug use phenotypes into general and drug-specific genetic (*a*), common environmental (*c*), and unique environmental factors (*e*).

**Results.:**

An IPM with one general *a* and one general *e* factor and a one-factor CPM provided comparable fit to the data. General factors accounted for 55% (*a* = 14%, *e* = 41%) and 79% (*a* = 64%, *e* = 15%) of the respective variation in POM and heroin use in the IPM, and 25% (*a* = 12%, *c* = 8%, *e* = 5%) and 80% (*a* = 38%, *c* = 27%, *e* = 15%) of the respective variation in POM and heroin use in the CPM. Across both models, POM emerged with substantial drug-specific genetic influence (26–39% of total phenotypic variance; 69–74% of genetic variance); heroin use did not (0% of total phenotypic variance; 0% of genetic variance in both models). Prescription sedative misuse also demonstrated significant drug-specific genetic variance.

**Conclusions.:**

Genetic variation in POM, but not heroin use, is predominantly drug-specific. Misuse of prescription medications that reduce experiences of subjective distress may be partially influenced by sources of genetic variation separate from illicit drug use.

Opioid use and related mortality remain a major public health concern (Substance Abuse and Mental Health Services Administration, 2021). Despite substantial effort to explicate the genetic etiology of opioid use and associated sequalae (Cheng et al., 2020; Deak et al., 2022; Jensen, 2016; Zhou et al., 2020), many twin and genomic studies have struggled to identify significant and/or replicable findings (Crist, Reiner, & Berrettini, 2019; Polimanti et al., 2020; Reed & Kreek, 2021). Heritability estimates of opioid use derived from twin studies have been varied, ranging from 0% to 79% (Dash, Martin, Agrawal, Lynskey, & Slutske, 2022; Karkowski, Prescott, & Kendler, 2000; Kendler, Aggen, Tambs, & Reichborn-Kjennerud, 2006; Kendler, Jacobson, Prescott, & Neale, 2003; Kendler, Karkowski, Neale, & Prescott, 2000a; Kendler, Karkowski, & Prescott, 1999; Tsuang et al., 1998; Van den Bree, Johnson, Neale, & Pickens, 1998). Such discrepancies in findings regarding the relative influence of genes and environment on opioid use may be at least partially attributable to the operationalization of opioid use as a single behavior encompassing both prescription opioid misuse (POM) and heroin use: emerging evidence suggests that POM and heroin use may be differentially influenced by genes and environment, such that POM is more strongly influenced by genes and heroin use is more strongly influenced by common environment (Dash et al., 2022; Gillespie et al., 2019).

Multivariate twin studies have demonstrated that liability for drug use can be accounted for by both a general propensity for any drug use and some amount of drug-specific influence, though most, if not all, such studies have utilized an aggregated opioid use phenotype. Genetic and common environmental influence on opioid use appear to be attributable to liability shared with cannabis, cocaine, hallucinogen, sedative, and stimulant use, though the magnitude of genetic and common environmental influence on opioid use identified in these studies is varied (3–37% and 17–40%, respectively); there also appears to be opioid-specific unique environmental influence (13–29% of the phenotypic variance) (Karkowski et al., 2000; Kendler et al., 2003). Conversely, a DSM-III opioid abuse phenotype was found to have substantial drug-specific genetic influence (38% of the phenotypic variance) as well as drug-specific unique environmental influence (12% of the phenotypic variance) (Tsuang et al., 1998). It remains unclear whether these patterns of findings would hold for POM and heroin use individually.

Multivariate twin studies have also identified two distinct sets of genetic risk factors for substance use, with one predisposing to licit use (alcohol, caffeine, nicotine), the other to illicit use (cannabis, cocaine), and some degree of shared genetic risk across these two factors (Kendler, Myers, & Prescott, 2007). It is not immediately obvious whether prescription misuse behaviors are more aptly operationalized as ‘licit’ or ‘illicit’ use given that prescription drugs are conditionally legal to obtain and possess, but can be acquired illegally and/or used in a manner more consistent with illicit drug use behavior (e.g. via insufflation, injection). Substantial evidence indicates that individuals reporting POM tend to misuse other prescription drugs at higher rates, whereas individuals reporting heroin use tend to use other illicit drugs at higher rates (Dash, Martin, Agrawal, Lynskey, & Slutske, 2021a; Rigg & Monnat, 2015; Wu, Woody, Yang, & Blazer, 2011). In other words, POM behavior tends to ‘cluster’ more closely with other prescription misuse behaviors (sedative, tranquilizer), while heroin use tends to ‘cluster’ more closely with use of other illicit drugs (cocaine, inhalants, hallucinogens), supporting the notion of distinct but correlated licit/prescription and illicit (mis)use factors.

## Present study

To date, twin studies that have parsed POM and heroin use have only done so within univariate and bivariate frameworks (Dash et al., 2022; Gillespie et al., 2019). The present study aimed to expand on this emergent literature by modeling POM and heroin use as distinct phenotypes within a multivariate framework as a means of explicating the degree to which their genetic and environmental influences are shared both with each other and with other drug (mis)use more broadly. Further, we aimed to explore the notion of separable prescription and illicit (mis)use factors by modeling POM and heroin use on distinct genetic and environmental factors alongside other forms of prescription misuse and illicit drug use, respectively. It was hypothesized that such a configuration would provide a better explanatory model than a single factor model, thereby replicating prior findings of differentiable sources of genetic influence across subcategories of substances (Kendler et al., 2007).

## Methods

### Participants and procedure

Data were drawn from Australian Twin Registry (ATR) cohorts II and III, which include Australian twins of primarily European ancestry born between 1964 and 1979. ATR cohort II data were collected between 1996 and 2000 via a telephone interview; ATR cohort III data were collected between 2005 and 2009 via computer-assisted telephone interview. The combined sample was comprised of 7164 individual twins [mean age = 30.58 years (s.d. = 2.64), range = 22–43] from both complete and incomplete same-sex pairs [monozygotic (MZ) male = 1555; MZ female = 2405; dizygotic (DZ) male = 1324; DZ female = 1880]. Of the full sample, 6026 individual twins (84.12%) were part of a complete twin pair (MZ male = 1264; MZ female = 2122; DZ male = 1030; DZ female = 1610).

### Measures

Assessments were conducted within the Semi-Structured Assessment for the Genetics of Alcoholism, adapted for the Australian population (SSAGA-OZ) (Bucholz et al., 1994). Ahead of their interview, participants were provided with a respondent booklet containing lists of specific drugs described by name and by common slang terms, where relevant. Lists were grouped by drug class, and included cannabis/hashish, cocaine/crack, amphetamine-based stimulants, opioids, sedatives, hallucinogens, dissociatives, solvents, and inhalants. Participants were asked ‘Have you ever used any of the items in List [X]?’ A positive response prompted a query as to which drug(s) on the list they had used, with instruction for reporting misuse of medically indicated drugs (‘when not prescribed or more than prescribed’). Responses were coded as binary (yes/no) variables.

### Analytic plan

Analyses were conducted in Mplus Version 8 (Muthén, 2017). All models were fitted by the method of robust weighted least squares directly to the raw twin data, which uses data from incomplete as well as complete twin pairs, and bias-corrected bootstrapped confidence intervals were estimated. A liability-threshold model, which assumes that there is a latent liability continuum underlying the binary drug use variables, was employed (Neale & Cardon, 2013). Thresholds and variances were constrained to equality across twins and zygosity groups but were permitted to differ across men and women after testing against saturated models determined that these specifications fit the data well. Univariate biometric models were run to partition the variation in drug use liability into additive genetic (*a*), common environmental (*c*), and unique (individual-specific) environmental (*e*) influences; the latter also includes measurement error. Quantitative sex differences were examined via Wald tests to determine if the magnitude of *a*, *c*, and *e* influences differed across men and women. A series of bivariate biometric models were then fit to estimate the phenotypic and cross-twin cross-trait correlations between POM and heroin use, POM and all other drug use phenotypes, and heroin use and all other drug use phenotypes.

Next, a series of independent pathway models (IPMs) and common pathway models (CPMs) were run. IPMs estimate general *a*, *c*, and *e* factors that influence all phenotypes, as well as drug-specific (residual) *a*, *c*, and *e* effects. The IPM assumes that *a*, *c*, and *e* factors operate independently of one another to influence manifest phenotypes (see online Supplementary Fig. S1). Congruent with other studies applying IPMs to substance use phenotypes (Kendler et al., 2003), and determined to be appropriate after testing against a saturated model, [χ^2^(399) = 424.56, *p* = 0.18], we began with a model comprised of two general *a* factors, two general *c* factors, and two general *e* factors, as well as drug-specific *a*, *c*, and *e* factors for each phenotype, here referred to as the ‘2–2-2 model’ wherein each number corresponds to the number of general *a*-*c*-*e* factors in the model. To test the hypothesis of separable genetic and environmental influences on prescription misuse and illicit use, constraints were imposed such that prescription opioid, sedative, and stimulant use loaded on one of the two factors for a given variance component, while heroin, cannabis, cocaine/crack, illicit stimulant, hallucinogens, inhalant, solvent, and dissociative use loaded on the other. This configuration was imposed iteratively upon each variance component in combination and alone (i.e. *ace*, *ac*, *ae*, *ce*, *a*, *c*, *e*) within the full 2–2-2 model, and model comparison was conducted via Wald tests. We subsequently conducted further testing of reduced models to identify the optimal number of factors and determine the most parsimonious solution. The CPM was used as a comparator against the IPM. The CPM assumes that the covariation between phenotypes is attributable to the influence of an intermediate, latent general phenotype that combines the effects of *a*, *c*, and *e* such that they do not act independently (see online Supplementary Fig. S2). As such, the *a*, *c*, and *e* effects mediated via the general phenotype are proportionally equivalent across all observed phenotypes in the model. In addition to the general phenotype, drug-specific *a*, *c*, and *e* influences were modeled within the CPM. Model comparison was conducted to identify the best-fitting and most parsimonious model via Wald tests. Sex-specific effects were not explored in the IPMs and CPMs to enhance power by utilizing the full combined sample, though models with sex included as a covariate were run. Model fit was evaluated via the root mean square error of approximation (RMSEA) (Steiger, 1990), the non-normed fit index (i.e. Tucker–Lewis Index or TLI) (Tucker & Lewis, 1973) and the comparative fit index (CFI) (Bentler, 1990). Cutoffs were applied as follows: RMSEA < 0.05, TLI > 0.95, and CFI > 0.95 (Chen, Curran, Bollen, Kirby, & Paxton, 2008; Hooper, Coughlan, & Mullen, 2008; Hu & Bentler, 1999; Yu, 2002).

## Results

### Descriptive findings

Lifetime POM and heroin use were endorsed by 7.79% and 1.31% of the sample, respectively. Cannabis was the most commonly used drug (61.81%), followed by prescription stimulants (17.46%), hallucinogens (16.85%), illicit stimulants (15.05%), inhalants (13.31%), cocaine/crack (11.96%), prescription sedatives (8.94%), solvents (3.41%), and dissociatives (1.86%). Prevalence of (mis)use for each drug by zygosity group is presented in online Supplementary Table S1. Men misused prescription stimulants [χ^2^(1) = 88.78, *p* < 0.001] and used heroin [χ^2^(1) = 20.23, *p* < 0.001], cannabis [χ^2^(1) = 145.84, *p* < 0.001], cocaine/crack [χ^2^(1) = 52.60, *p* < 0.001], illicit stimulants [χ^2^(1) = 33.74, *p* < 0.001], hallucinogens [χ^2^(1) = 114.45, *p* < 0.001], inhalants [χ^2^(1) = 130.07, *p* < 0.001], solvents [χ^2^(1) = 22.80, *p* < 0.001], and dissociatives [χ^2^(1) = 19.38, *p* < 0.001] at higher rates than women; only prescription opioid and sedative misuse did not differ in rates of use across men and women (*p*s = 0.41–0.57).

**Fig. 1.**
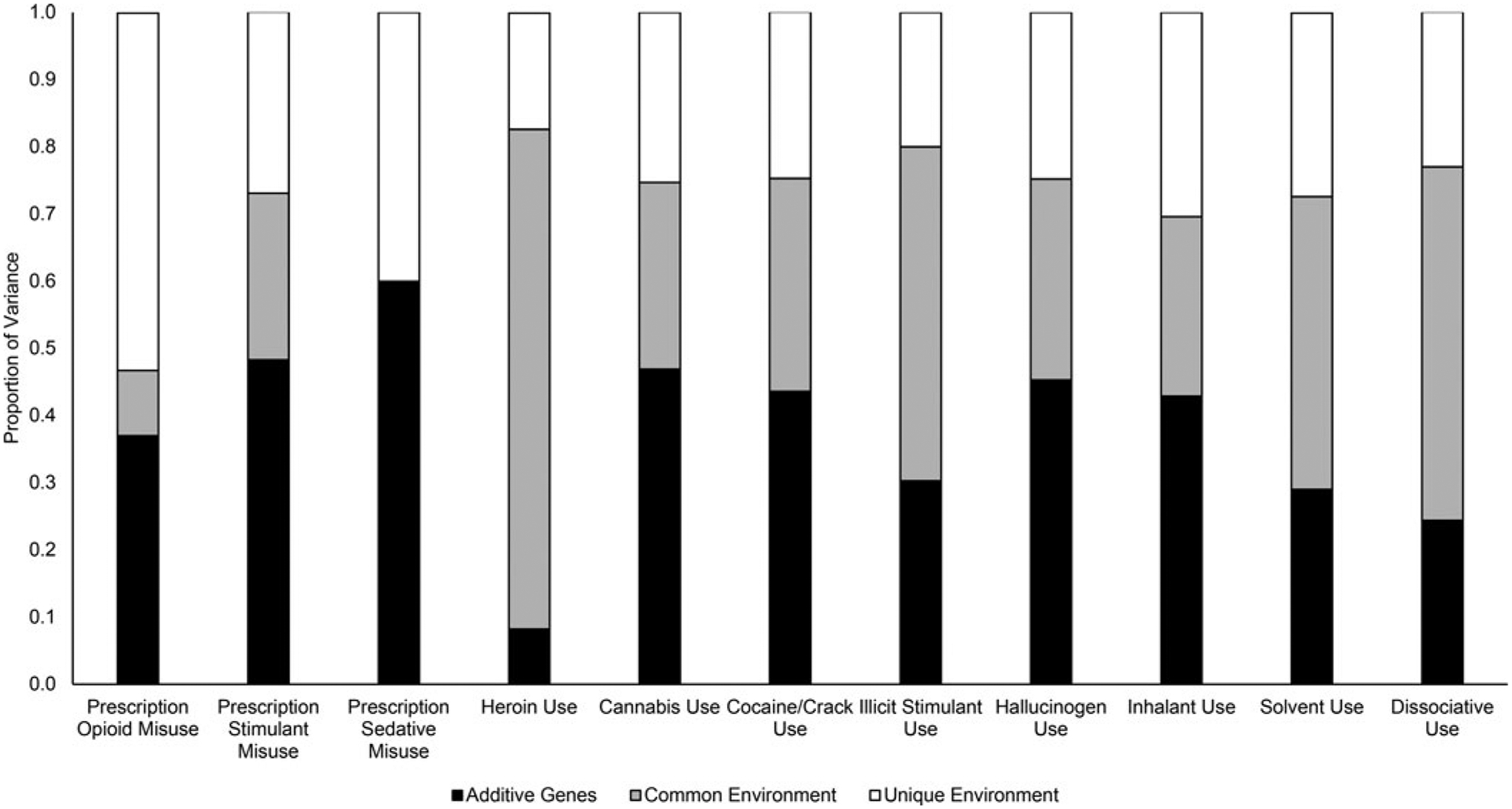
Proportion of variance in lifetime drug use attributable to additive genetic (*a*), common environmental (*c*), and unique environmental (*e*) factors in univariate models. *Note*: Parameters could be constrained to equality across men and women in all models except cannabis use, for which *a* and *c* parameters could be individually, but not simultaneously, constrained [Wald χ^2^(2) = 8.02, *p* = 0.02]; *a*, *c*, and *e* estimates from the freely estimated model were 31, 33, and 37% for men and 51, 27, and 22% for women (estimates from the constrained cannabis use model are presented for consistency).

Variance estimates from univariate models are depicted in Fig. 1 (standardized coefficients, confidence intervals, and model fit statistics are available in online Supplementary Table S2). Estimates for *a*, *c*, and *e* components could be constrained to equality across men and women for all drug use phenotypes except cannabis use. POM was attributable to *a* (37%), *c* (10%, *ns*), and *e* (53%); heroin use was attributable to *a* (8%, *ns*), *c* (74%), and *e* (17%). For the remaining drugs, the proportion of *a* influence ranged from 24% (*ns*) for dissociative use to 60% for prescription sedative misuse; the proportion of *c* influence ranged from 0% for prescription sedative misuse to 53% for dissociative use; and the proportion of *e* influence ranged from 20% for illicit stimulant use to 40% for prescription sedative misuse.

Bivariate correlations are presented in Table 1. Cannabis use represented the weakest association for both opioid types in the phenotypic (within-twin) correlations. POM was most strongly associated with prescription sedative misuse and heroin use among both men (*r* = 0.70–0.65) and women (*r* = 0.55–0.64). Heroin use was most strongly associated with cocaine/crack use (*r* = 0.81) and hallucinogen use among men (*r* = 0.75) and hallucinogen use (*r* = 0.81), cocaine/crack use (*r* = 0.80), and dissociative use (*r* = 0.80) among women. For the cross-twin cross-trait correlations, MZ twin correlations were generally larger in magnitude than the DZ correlations, implying some degree of genetic influence on the overlap between POM, heroin use, and other drug use. Cross-twin cross-trait correlations were substantially larger in magnitude for heroin and other drug use than for POM and other drug use.

### Multivariate models

#### Model selection

Results of model comparison are presented in Table 2. The full 2–2-2 IPM, comprised of two general factors for each *a*, *c*, and *e* as well as drug-specific *a*, *c*, and *e* factors, provided good fit to the data [χ^2^(399) = 424.58; RMSEA = 0.006 (95% CI 0.000–0.010); TLI = 0.999; CFI = 0.999]. Although the prescription-illicit factor configuration could be imposed upon each variance component alone and in combination without worsening model fit (see online Supplementary Table S3), further model comparison indicated that this was likely due to two general factors per variance component being unnecessary. That is, the second general *a*, *c*, and *e* factors could all be dropped without significant decrement in model fit [Wald χ^2^(30) = 13.16, *p* = 0.99] to create a 1-1-1 model with only one general factor for each *a*, *c*, and *e*, suggesting that variance shared across drugs is common to all drug types rather than separable clusters of drugs (i.e. prescription and illicit). While dropping any one of the general factors from the resulting 1–1-1 IPM did not significantly worsen model fit, the model with no general *a* factor (0–1-1 model) and the model with no general *e* factor (1–1-0 model) did not converge, implying poor fit of these models to the data. As such, we proceeded with a model with one general *a* and one general *e* factor (1–0-1 model), which did not worsen model fit compared to either the 2–2-2 [Wald χ^2^(41) = 15.01, *p* = 0.99] or 1–1-1 IPMs [Wald χ^2^(11) = 0.69, *p* > 0.99]. This 1–0-1 model with one general *a* and one general *e* factor could be further reduced by dropping non-significant parameters, which included the loading for cannabis use on the general *e* factor; drug-specific *a* factors for prescription stimulant misuse and all illicit drug use phenotypes; and drug-specific *c* factors for all prescription misuse phenotypes and for heroin, cocaine/crack, hallucinogen, and dissociative use [Wald χ^2^(17) = 19.80, *p* = 0.28]. However, drug-specific *a* and *c* factors could not be completely dropped from the model [Wald χ^2^(22) = 98.42, *p* < 0.001], confirming appreciable magnitude of drug-specific effects. Drug-specific *e* factors were retained for all phenotypes, as it is unlikely that they were measured without error. The reduced 1–0-1 model provided good fit to the data [χ^2^(457) = 794.97; RMSEA = 0.019 (95% CI 0.017–0.021); TLI = 0.990; CFI = 0.990].

A one-factor CPM also provided good fit to the data [χ^2^(438) = 761.05; RMSEA = 0.019 (95% CI 0.017–0.021); TLI = 0.991; CFI = 0.990]. All non-significant parameters could be dropped without significant decrement in model fit [Wald χ^2^(11) = 1.64, *p* = 0.99]; this included drug-specific *a* factors for heroin, cocaine/crack, illicit stimulant, hallucinogen, solvent, and dissociative use, and drug-specific *c* factors for prescription opioid, stimulant, and sedative misuse, cannabis use, and inhalant use. Drug-specific *a* and *c* factors could not be completely dropped from the model [Wald χ^2^(22) = 405.27, *p* < 0.001], again confirming appreciable magnitude of drug-specific effects. As in the IPM, drug-specific *e* factors were retained for all phenotypes. The reduced CPM fit the data well [χ^2^(449) = 766.42; RMSEA = 0.018 (95% CI 0.016–0.021); TLI = 0.991; CFI = 0.990].

Overall, the reduced 1-0-1 IPM and the reduced one-factor CPM provided comparable fit to the data, with nearly identical RMSEA, TLI, and CFI values. Model preference in this case is not clear. It has been argued that the IPM is the superior model, given that it makes fewer assumptions (Kendler et al., 2003); conversely, it has been argued that the CPM is the superior model, as it estimates fewer parameters and is therefore more parsimonious (Tsuang et al., 1998). As such, both models are presented below.

#### Model results

##### Independent pathway model.

**Fig. 2.**
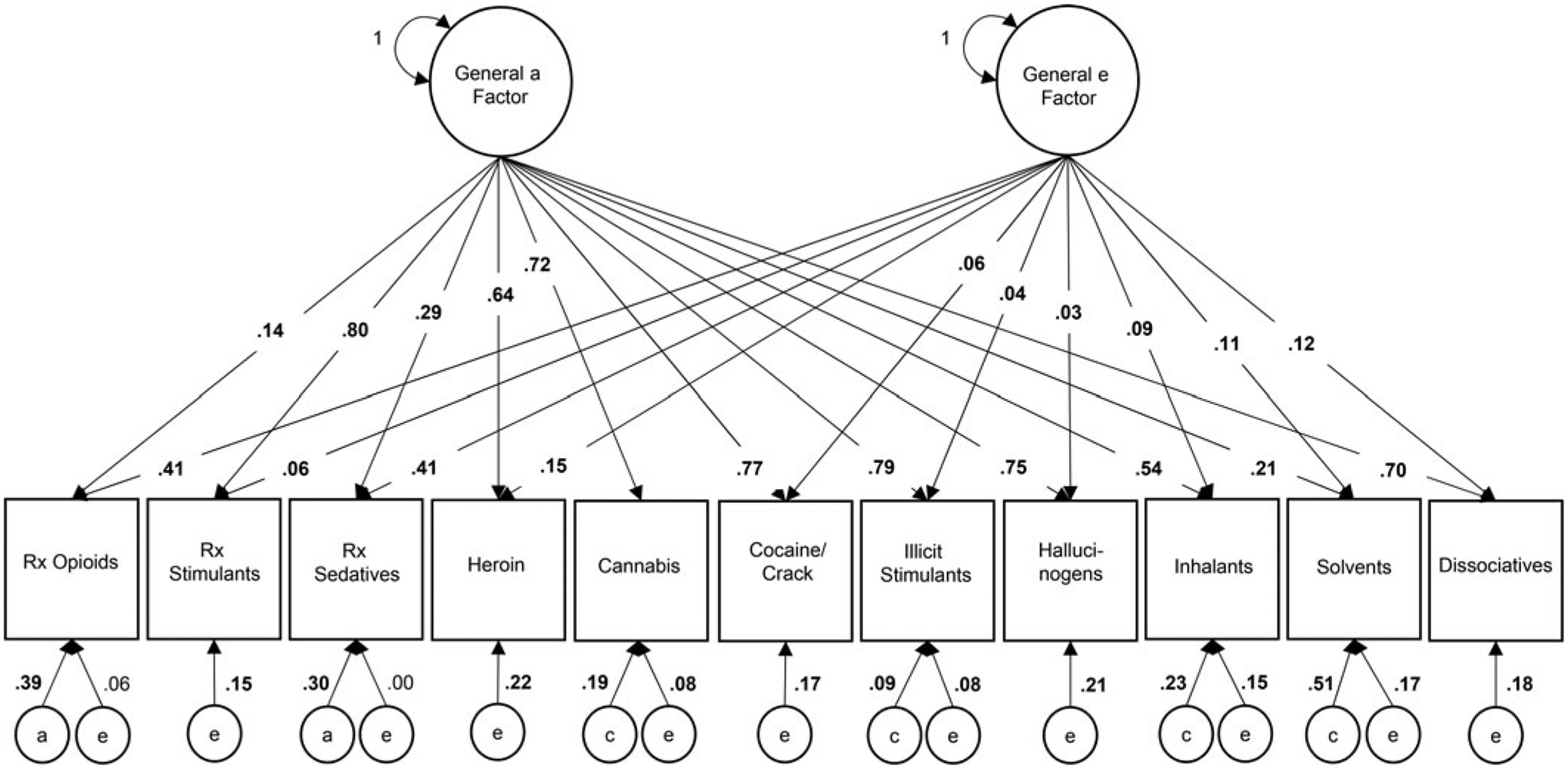
Proportion of variance in lifetime drug use attributable to general and drug-specific additive genetic (*a*), common environmental (*c*), and unique environmental (*e*) factors in the best-fit independent pathway model. *Note*: Bold font indicates significant parameter estimate, *p* < 0.001; variance components may not sum to 1 due to rounding error; variances of residual components were set to 1 (not depicted).

Variance estimates from the best-fit IPM (reduced 1-0-1), directly interpretable as the proportion of variance in each manifest trait attributable to each factor, are depicted in Fig. 2 (standardized coefficients and confidence intervals are available in online Supplementary Table S4). For POM, 55% of the variance was attributable to general factors, primarily through the general *e* factor (41% of the phenotypic variance); for heroin use, 79% of the variance was attributable to general factors, primarily through the general *a* factor (64% of the phenotypic variance). The general *a* factor contributed significantly to the total variance in all drug use phenotypes (14–80%), most weakly for POM (14%) and most strongly for prescription stimulant misuse (80%). Drug-specific *a* influence emerged only for POM (39% of the phenotypic variance) and prescription sedative misuse (30% of the phenotypic variance); the drug-specific *a* parameter for all other phenotypes, including heroin use, could be constrained to 0. Drug-specific *c* influences ranged from 9% of the phenotypic variance for illicit stimulant use to 51% of the phenotypic variance for solvent use. The general *e* factor contributed substantially to the total variance in POM (41%) and sedative misuse (41%), and modestly to the total variance in remaining phenotypes (3–15%). Drug-specific *e* influences (and error) also contributed modestly to all phenotypes (6–22%) except prescription sedative misuse (<1%).

##### Common pathway model.

**Fig. 3.**
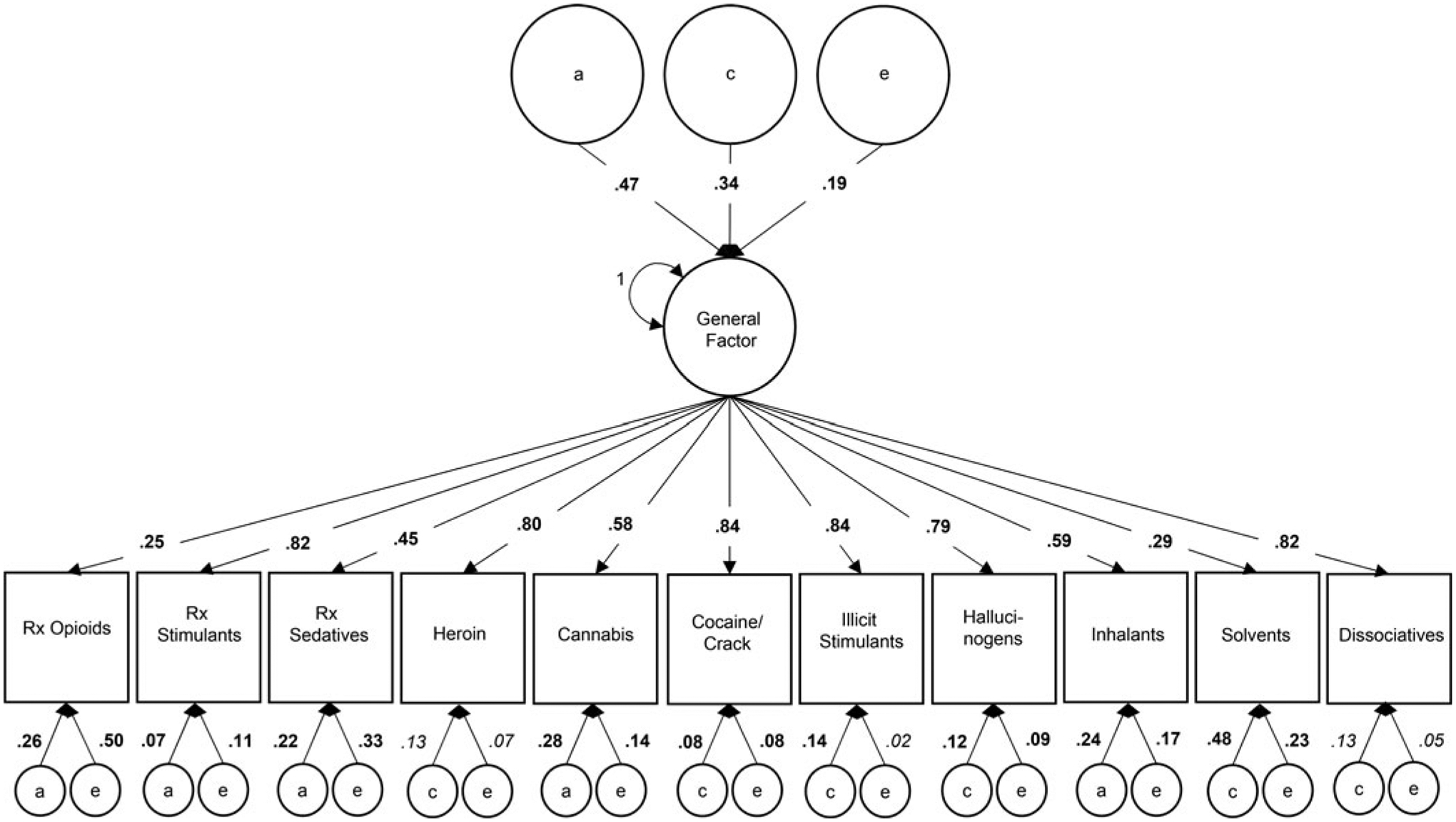
Proportion of variance in lifetime drug use attributable to a latent general factor and drug-specific additive genetic (*a*), common environmental (*c*), and unique environmental (*e*) factors in the best-fit common pathway model. *Note*: Bold font indicates significant parameter at *p* < 0.001; italic font indicates significant parameter at *p* < 0.05; variance components may not sum to 1 due to rounding error; variances of residual components were set to 1 (not depicted).

Variance estimates from the best-fit one-factor CPM, again directly interpretable as the proportion of variance accounted for, are depicted in Fig. 3 (standardized coefficients and confidence intervals are available in online Supplementary Table S5). Variation in the general phenotypic factor was 47% attributable to *a*, 34% attributable to *c*, and 19% attributable to *e*. Variation in drug (mis)use phenotypes attributable to the general phenotypic factor ranged from 25% for POM to 84% for cocaine/crack and illicit stimulant use; 80% of the phenotypic variance in heroin use was attributable to the general factor. The remaining variance in POM was attributable to drug-specific *a* (26%) and *e* (50%) influences; the remaining variance in heroin use was attributable to modest drug-specific effects of *c* (13%) and *e* (7%). Drug-specific *a* influences also emerged for prescription sedative misuse (22%), cannabis use (28%), and inhalant use (24%). Consistent with the IPM, drug-specific *c* influences only emerged for illicit drug use phenotypes (8–48%), including a modest contribution for heroin use (13%). Drug-specific *e* influences again contributed modestly to most drug use (2–23%), including heroin use (7%), but quite substantially for POM (50%) and prescription sedative misuse (33%).

### Supplemental models

Because prevalence rates across most drug (mis)use phenotypes differed across men and women, supplementary IPM and CPM analyses with sex included as a covariate were run. Variance estimates are presented in online Supplementary Table S6. Sex accounted for as little as 0% of the variance (POM, prescription sedative misuse) and at most 5% of the variance (inhalant use) in drug (mis)use phenotypes across models. The overall pattern of results was essentially unchanged.

## Discussion

The present study aimed to explore specificity in genetic and environmental risk for POM and heroin use within a multivariate twin framework. Most twin studies using this approach have utilized an aggregate opioid use phenotype that combines POM and heroin use, potentially obscuring unique influences on each opioid type (Dash et al., 2022; Karkowski et al., 2000; Kendler et al., 1999, 2003; Tsuang et al., 1998). Here, we aimed to identify whether and to what degree POM and heroin use are influenced by distinct etiologic influences and to explore how genetic and environmental influences on POM and heroin use are situated within a broader constellation of drug (mis)use behaviors. Results did not provide definitive support for the IPM *v*. the CPM, which is consistent with mixed findings regarding the relative fit of these models in multivariate twin studies of drug use (Kendler et al., 2003; Tsuang et al., 1998). However, the conclusions regarding general and specific genetic and environmental influences on POM and heroin use were quite similar across the IPM and CPM. Though their assumptions regarding the nature of shared influences differ, both models identified substantial drug-specific additive genetic variance in POM (26–39% of the total phenotypic variance; 69–71% of the total genetic variance) that was not similarly present for heroin use (0% of the total phenotypic variance in both models; 0% of the total genetic variance in both models), the latter of which was primarily accounted for by variance shared with other drug use (79–80% of the total phenotypic variance). This supports past findings of smaller genetic and environmental correlations between POM and heroin use than would perhaps be expected (Dash et al., 2022).

Also notable were the patterns of effect for prescription sedative misuse, which largely mirrored those of POM. Both POM and prescription sedative misuse emerged with relatively low loadings on general factors shared across drugs and more robust drug-specific influences (45–76% and 30–55% of the total phenotypic variance in POM and prescription sedative misuse, respectively). Such findings suggest that, despite a lack of support for differentiable prescription and illicit use factors, there is something unique about the etiology of prescription misuse behaviors, at least for drugs that act as ‘downers.’ It is not clear why prescription stimulant misuse did not adhere to this pattern, with only 18–21% of the phenotypic variance being drug-specific and predominantly attributable to unique environment. One reason may be differences in subjective drug effects that result in distinct instrumental reasons for misuse of prescription ‘downers’ such as opioids and sedatives *v*. prescription ‘uppers’ such as stimulants. For example, individual differences such as personality may facilitate use of particular drugs; studies have shown that neuroticism and hopelessness predict opioid and sedative use, while extraversion and sensation-seeking predict stimulant use (Dash, Martin, & Slutske, 2021b; Mahu et al., 2019). Prescription stimulants are also used more frequently in a manner consistent with illicit drug use (e.g. intranasal administration) (Butler et al., 2021; Wilens et al., 2008), which may indicate that prescription stimulant misuse aligns more closely with use of other illicit drugs than with other prescription misuse behaviors.

More broadly, the finding that variance shared across drugs could be modeled as a single factor, as opposed to smaller, separable clusters, is consistent with phenotypic and genotypic findings on the factor structure of substance use and use disorder – and psychopathology more broadly – that have identified an overarching factor accounting for overlap across phenotypes (Caspi et al., 2014; Caspi & Moffitt, 2018; Hatoum et al., 2022; Hicks, Schalet, Malone, Iacono, & McGue, 2011; Sanchez-Roige, Kember, & Agrawal, 2022). These findings are also consistent with a past twin study of drug use that found genetic variance shared between opioids, sedatives, cannabis, cocaine, stimulants, and hallucinogens to be captured by a single factor (Kendler et al., 2003). Though a twin study testing licit and illicit factors conducted by Kendler et al. (2007) identified a licit factor comprised of alcohol, caffeine, and nicotine dependence symptoms and an illicit factor comprised of cannabis and cocaine dependence symptoms, there are plausible explanations as to why this two-factor pattern did not replicate for the prescription-illicit configuration tested here. Alcohol, caffeine, and nicotine are more widely available, more commonly used, and, among adults, universally legal in the place and time of data collection (Australian Department of Health and Aged Care, 2021; Australia Department of Health and Aged Care, 2022; SAMHSA, 2021). These characteristics of ‘licit use’ as operationalized by Kendler et al. (2007) may have contributed to a starker contrast between licit and illicit use than would be observed between prescription misuse and illicit use. That is, differences between prescription misuse and illicit use may be more subtle so as to be insufficient to form clearly separable factors. However, the pattern of results for POM and prescription sedative misuse suggests that the present study may have been underpowered to detect the uniqueness of the prescription misuse phenotypes. Future research in larger datasets may be equipped to shed further light on this pattern.

Overall, the finding that POM may share little genetic influence with heroin use has several implications for opioid research and policy. First, such a pattern suggests that disaggregating POM and heroin use could increase the replicability of findings across samples with varying proportions of POM *v*. heroin use (Cheng et al., 2018; Dash et al., 2022). Second, the presence of POM-specific genetic influence unshared with heroin use has important implications for case–control selection in genomic studies of opioid use disorder (OUD), which typically do not differentiate POM-exposed individuals from heroin-exposed individuals, nor POM- *v*. heroin-based OUD (Cheng et al., 2018; Dash et al., 2022; Gelernter et al., 2014; Zhou et al., 2020). This is also salient considering that heroin-exposed individuals tend to progress to OUD at higher rates than POM-exposed individuals (Wu et al., 2011). Thus, POM- and heroin-involved individuals could be differentially represented among cases and exposed controls, thereby introducing potential uncontrolled confounds into such studies. Third, if POM and heroin use have little etiologic overlap, public health efforts seeking to mitigate the impact of heroin and other illicit opioid use by reducing opioid prescribing rates are unlikely to be an optimal approach. Indeed, overdose deaths due to opioids have not dropped in parallel with declines in opioid prescribing, and there are increasing calls to shift efforts toward addressing the root social and economic causes of the opioid crisis (Dasgupta, Beletsky, & Ciccarone, 2018; Mattson et al., 2021).

### Limitations

The results of the present study should be interpreted in light of limitations. First, it is not clear how findings from this Australian sample will generalize to other countries and cultures. The sample was comprised primarily of individuals of European ancestry and data on self-identified race and ethnicity were not available. Additionally, the data collection period for a subset of the present sample occurred very early in the rise of the global opioid crisis, potentially limiting generalizability to the contemporary public health landscape. It is also important to note here that heritability estimates are specific to a particular population at a given point in time; it will be important to collect novel data so as to examine this topic in more recent samples, as evidence suggests that heritability of substance use can vary over time as a function of environmental factors such as changes in drug accessibility (Boardman, 2009; Slutske, Deutsch, & Piasecki, 2019a; Slutske, Piasecki, Deutsch, Statham, & Martin, 2019b) and social acceptability (Kendler, Thornton, & Pedersen, 2000b; Mezquita et al., 2018). Second, we were unable to conduct multivariate models in sex-specific zygosity groups due to sparse endorsement of the phenotypes of interest, though supplemental models revealed minimal differences between models with and without sex included as a covariate. Similarly, given low endorsement of heroin use in particular, the present analyses may have been underpowered. There were more illicit use phenotypes than prescription misuse phenotypes and therefore an imbalance in the number of indicators per factor in models with a two-factor configuration, which may have also impacted model fitting. Finally, we were restricted to binary indicators of lifetime drug use. Despite these limitations, the present study provides novel insight into the specificity (or lack thereof) in genetic and environmental influences on POM and heroin use, as well as several other drug (mis)use behaviors.

## Conclusions

POM and heroin use are often aggregated into a single ‘opioid use’ variable, but the global health burden of opioid consumption has highlighted the potential need for examining these behaviors as distinct phenotypes. The present study suggests that POM is non-negligibly attributable to additive genetic influences not shared with heroin use or use of other illicit drugs. Behavior genetic research, and opioid research more broadly, may consider implementing finer-grained assessment and operationalization of opioid use, including deep phenotyping, that can more effectively and consistently differentiate POM and heroin use behaviors, as well as different manifestations of such behaviors (e.g. more than prescribed *v*. when not prescribed), so as to avoid overlooking potentially important characteristics unique to each opioid form and the manner in which they are used. Doing so may have public health implications of critical importance germane to effectively addressing the opioid crisis via public policy, and may help to explain the underwhelming effectiveness of opioid prescribing restrictions on reducing the population-level impact of opioid use and related mortality.

## Supplementary Material

Supplement

## Figures and Tables

**Table 1.** Cross-trait correlations for prescription opioid misuse, heroin use, and other drug (mis)use derived from bivariate biometric models

		Prescription opioid misuse		Heroin use	
		Within-twin	Cross-twin	Within-twin	Cross-twin
Phenotype		*r* (95% CI)	*r* (95% CI)	*r* (95% CI)	*r* (95% CI)
Prescription opioid misuse	MZM	–	–	**0.64 (0.52–0.75)**	**0.53 (0.32–0.69)**
	MZF	–	–	**0.64 (0.49–0.75)**	**0.53 (0.34–0.69)**
	DZM	–	–	**0.65 (0.52–0.75)**	**0.27 (0.14–0.37)**
	DZF	–	–	**0.64 (0.49–0.75)**	**0.41 (0.23–0.58)**
Prescription stimulant misuse	MZM	**0.52 (0.44–0.59)**	**0.36 (0.23–0.49)**	**0.72 (0.65–0.78)**	**0.62 (0.50–0.73)**
	MZF	**0.39 (0.32–0.46)**	**0.30 (0.19–0.40)**	**0.78 (0.72–0.83)**	**0.67 (0.51–0.78)**
	DZM	**0.52 (0.44–0.59)**	**0.22 (0.11–0.34)**	**0.72 (0.65–0.78)**	**0.36 (0.23–0.51)**
	DZF	**0.39 (0.32–0.46)**	**0.21 (0.10–0.33)**	**0.78 (0.72–0.83)**	**0.53 (0.39–0.69)**
Prescription sedative misuse	MZM	**0.70 (0.63–0.76)**	**0.46 (0.32–0.59)**	**0.69 (0.57–0.77)**	**0.61 (0.41–0.76)**
	MZF	**0.55 (0.48–0.61)**	**0.31 (0.19–0.44)**	**0.62 (0.49–0.74)**	**0.61 (0.43–0.73)**
	DZM	**0.70 (0.63–0.76)**	**0.23 (0.14–0.33)**	**0.69 (0.57–0.77)**	**0.30 (0.18–0.41)**
	DZF	**0.55 (0.48–0.61)**	*0.15* (*0.05–0.27*)	**0.62 (0.49–0.74)**	**0.47 (0.33–0.63)**
Heroin use	MZM	**0.65 (0.52–0.75)**	**0.53 (0.32–0.69)**	–	–
	MZF	**0.64 (0.49–0.75)**	**0.53 (0.34–0.69)**	–	–
	DZM	**0.65 (0.52–0.75)**	**0.27 (0.14–0.37)**	–	–
	DZF	**0.64 (0.49–0.75)**	**0.41 (0.23–0.58)**	–	–
Cannabis use	MZM	**0.25 (0.15–0.35)**	**0.23 (0.10–0.35)**	**0.45 (0.40–0.50)**	**0.38 (0.19–0.53)**
	MZF	**0.25 (0.18–0.32)**	**0.17 (0.08–0.27)**	**0.46 (0.38–0.52)**	**0.44 (0.32–0.58)**
	DZM	**0.25 (0.15–0.35)**	**0.26 (0.17–0.39)**	**0.45 (0.40–0.50)**	**0.29 (0.12–0.41)**
	DZF	**0.25 (0.18–0.32)**	**0.23 (0.13–0.31)**	**0.46 (0.38–0.52)**	**0.35 (0.19–0.47)**
Cocaine/crack use	MZM	**0.56 (0.48–0.63)**	**0.38 (0.25–0.54)**	**0.81 (0.74–0.86)**	**0.57 (0.38–0.73)**
	MZF	**0.32 (0.22–0.41)**	**0.26 (0.12–0.40)**	**0.80 (0.70–0.86)**	**0.61 (0.47–0.74)**
	DZM	**0.56 (0.48–0.63)**	*0.18* (*0.06–0.29*)	**0.81 (0.74–0.86)**	**0.30 (0.16–0.43)**
	DZF	**0.32 (0.22–0.41)**	**0.28 (0.15–0.39)**	**0.80 (0.70–0.86)**	**0.59 (0.45–0.72)**
Illicit stimulant use	MZM	**0.49 (0.41–0.56)**	**0.38 (0.25–0.50)**	**0.70 (0.59–0.77)**	**0.54 (0.36–0.71)**
	MZF	**0.33 (0.26–0.41)**	**0.22 (0.10–0.34)**	**0.78 (0.70–0.84)**	**0.62 (0.44–0.74)**
	DZM	**0.49 (0.41–0.56)**	**0.29 (0.17–0.40)**	**0.70 (0.59–0.77)**	**0.47 (0.30–0.62)**
	DZF	**0.33 (0.26–0.41)**	**0.24 (0.12–0.34)**	**0.78 (0.70–0.84)**	**0.56 (0.41–0.71)**
Hallucinogen use	MZM	**0.47 (0.39–0.55)**	**0.39 (0.27–0.50)**	**0.75 (0.69–0.82)**	**0.60 (0.45–0.73)**
	MZF	**0.33 (0.24–0.41)**	**0.24 (0.11–0.36)**	**0.81 (0.74–0.86)**	**0.70 (0.55–0.82)**
	DZM	**0.47 (0.39–0.55)**	**0.19 (0.08–0.29)**	**0.75 (0.69–0.82)**	**0.31 (0.19–0.43)**
	DZF	**0.33 (0.24–0.41)**	**0.23 (0.12–0.35)**	**0.81 (0.74–0.86)**	**0.57 (0.47–0.71)**
Inhalant use	MZM	**0.47 (0.38–0.55)**	**0.38 (0.24–0.49)**	**0.64 (0.53–0.73)**	**0.48 (0.28–0.64)**
	MZF	**0.38 (0.29–0.47)**	**0.29 (0.15–0.44)**	**0.71 (0.60–0.80)**	**0.62 (0.44–0.75)**
	DZM	**0.47 (0.38–0.55)**	**0.20 (0.08–0.32)**	**0.64 (0.53–0.73)**	**0.41 (0.27–0.55)**
	DZF	**0.38 (0.29–0.47)**	**0.24 (0.12–0.38)**	**0.71 (0.60–0.80)**	**0.45 (0.29–0.61)**
Solvent use	MZM	**0.42 (0.30–0.54)**	**0.40 (0.22–0.57)**	**0.44 (0.27–0.59)**	**0.47 (0.18–0.69)**
	MZF	**0.29 (0.15–0.44)**	*0.24* (*0.07–0.40*)	**0.59 (0.45–0.75)**	**0.57 (0.35–0.70)**
	DZM	**0.42 (0.30–0.54)**	*0.19* (*0.06–0.34*)	**0.44 (0.27–0.59)**	**0.26 (0.11–0.42)**
	DZF	**0.29 (0.15–0.44)**	0.15 (0.00–0.34)	**0.59 (0.45–0.75)**	**0.54 (0.33–0.70)**
Dissociative use	MZM	**0.64 (0.52–0.74)**	**0.48 (0.30–0.65)**	**0.67 (0.55–0.78)**	**0.67 (0.46–0.82)**
	MZF	**0.49 (0.33–0.64)**	*0.31* (*0.06–0.52*)	**0.80 (0.63–0.90)**	**0.81 (0.62–0.92)**
	DZM	**0.64 (0.52–0.74)**	**0.24 (0.07–0.37)**	**0.67 (0.55–0.78)**	**0.41 (0.25–0.62)**
	DZF	**0.49 (0.33–0.64)**	*0.24* (*0.07–0.45*)	**0.80 (0.63–0.90)**	**0.69 (0.53–0.83)**

MZM, monozygotic male; MZF, monozygotic female; DZM, dizygotic male; DZF, dizygotic female.

*Note*: Bold font indicates significant correlation, *p* < 0.001; italic font indicates significant correlation, *p* < 0.05.

**Table 2.** Fix indices for independent pathway (IP) and common pathway (CP) models of drug (mis)use

	Model fit						Test of parameter constraints		
Model comparisons	χ^2^	df	*p*	RMSEA	TLI	CFI	Wald χ^2^	df	*p*
Independent pathway (IP)									
IP 2-2-2	424.584	399	0.18	0.006 (0.000-0.010)	0.999	0.999	–	–	–
Step I (*v*. 2-2-2)									
IP 2-2-1				No convergence			9.285	10	0.50
IP 2-1-2				No convergence			0.452	10	>0.99
IP 1-2-2	432.560	409	0.20	0.005 (0.000–0.010)	0.999	0.999	0.340	10	>0.99
Step II (*v*. 1-2-2)									
IP 1-2-1	462.593	419	0.07	0.007 (0.000–0.011)	0.999	0.999	11.199	10	0.34
IP 1-1-2	467.440	419	0.05	0.007 (0.000–0.011)	0.999	0.998	0.489	10	>0.99
IP 0-2-2				No convergence			11.200	11	0.43
Step III									
IP 1-1-1	566.657	429	<0.001	0.012 (0.009–0.015)	0.996	0.996			
(*v*. 1-2-1)							0.839	10	0.99
(*v*. 1-1-2)							16.545	10	0.09
Step IV (*v*. 1-1-1)									
IP 0-1-1				No convergence			18.523	11	0.07
IP 1-0-1	778.506	440	<0.001	0.019 (0.017–0.021)	0.990	0.990	0.694	11	>0.99
IP 1-1-0				No convergence			8.584	11	0.66
Step V (*v*. 1-0-1)									
IP 0-0-1				No convergence			75.880	11	<0.001
IP 1-0-0	1018.495	451	<0.001	0.025 (0.023–0.027)	0.983	0.983	28.906	11	0.002
IP 1-0-1 (reduced)	794.969	457	<0.001	0.019 (0.017–0.021)	0.990	0.990	19.797	17	0.28
Common pathway (CP)									
CP-1	761.052	438	<0.001	0.019 (0.017–0.021)	0.991	0.990	–	–	–
CP-1 (reduced)	766.419	449	<0.001	0.018 (0.016–0.021)	0.991	0.990	1.642	11	0.99

*Note*: Numbers in IP model names correspond to the model’s number of general *a*-*c*-*e* factors; numbers in CP model names correspond to the number of intermediate, latent general factors.
