# Supplementary material for: Specificity in genetic and environmental risk for prescription opioid misuse and heroin use
Source: Psychol Med. Author manuscript; Available in PMC 2023 Oct 27. (PMC10514228; doi:10.1017/S003329172300034X)
Supplement: Supplement [file NIHMS1885804-supplement-Supplement.docx]

**Supplemental Materials**

for

**Specificity in Genetic and Environmental Risk**

**for Prescription Opioid Misuse and Heroin Use**

Genevieve F. Dash^1^, Ian R. Gizer^1^, Nicholas G. Martin^2^, & Wendy S. Slutske^3^

^1^Department of Psychological Sciences, University of Missouri, Columbia, MO, 65211, USA

^2^QIMR Berghofer, Brisbane, Queensland, 4006, Australia

^3^Department of Family Medicine and Community Health and Center for Tobacco Research and Intervention, University of Wisconsin, Madison, WI, 53711, USA

| Table S1  *Sample prevalence of substance use across zygosity groups* | | | | | |
| --- | --- | --- | --- | --- | --- |
| Phenotype | Total  (N=7,164) | MZM (n=1,555) | DZM (n=1,324) | MZF (n=2,405) | DZF (n=1,880) |
|  | n (%) | n (%) | n (%) | n (%) | n (%) |
| Prescription Opioid Misuse | 558 (7.79) | 129 (8.30) | 86 (6.50) | 202 (8.40) | 141 (7.50) |
| Prescription Stimulant Misuse | 1,251 (17.46) | 330 (21.22) | 321 (24.24) | 339 (14.10) | 261 (13.88) |
| Prescription Sedative Misuse | 644 (8.99) | 139 (8.94) | 126 (9.52) | 227 (9.44) | 152 (8.09) |
| Heroin Use | 94 (1.31) | 28 (1.80) | 31 (2.34) | 20 (0.83) | 15 (0.80) |
| Cannabis Use | 4,428 (61.81) | 1,062 (68.30) | 957 (72.28) | 1,335 (55.51) | 1,074 (57.13) |
| Cocaine/Crack Use | 689 (9.62) | 186 (11.96) | 179 (13.52) | 181 (7.53) | 143 (7.61) |
| Illicit Stimulant Use | 963 (13.44) | 234 (15.05) | 235 (17.75) | 278 (11.56) | 216 (11.49) |
| Hallucinogen Use | 992 (13.85) | 262 (16.85) | 289 (21.83) | 235 (9.77) | 206 (10.96) |
| Inhalant Use | 661 (9.23) | 207 (13.31) | 195 (14.73) | 150 (6.24) | 109 (5.80) |
| Solvent Use | 153 (2.14) | 53 (3.41) | 37 (2.79) | 37 (1.54) | 26 (1.38) |
| Dissociative Use | 97 (1.35) | 29 (1.86) | 31 (2.34) | 23 (0.96) | 14 (0.74) |
| Note. MZM=monozygotic male; MZF=monozygotic female; DZM=dizygotic male; DZF=dizygotic female. | | | | | |

| Table S2  *Standardized estimates of additive genetic (a), common environmental (c), and individual-specific (e) environmental factors in univariate models* | | | | | | |
| --- | --- | --- | --- | --- | --- | --- |
| Phenotype | Variance Component | | | Model Fit | | |
|  | *a* (95% CI) | *c* (95% CI) | *e* (95% CI) | χ^2^ | df | *p* |
| Prescription Opioid Misuse | **.61 (.02, .74)** | .31 (.00, .64) | **.73 (.65, .80)** | 13.93 | 8 | .08 |
| Prescription Stimulant Misuse | **.70 (.51, .84)** | **.50 (.23, .67)** | **.52 (.46, .57)** | 8.07 | 8 | .43 |
| Prescription Sedative Misuse | **.78 (.71, .83)** | .01 (.00, .01) | **.63 (.56, .70)** | 6.73 | 8 | .57 |
| Heroin Use | .29 (.00, .81) | **.86 (.58, .94)** | **.42 (.27, .59)** | 5.39 | 8 | .72 |
| Cannabis Use | **.69 (.56, .80)** | **.53 (.35, .65)** | **.50 (.47, .55)** | 21.02 | 8 | .007 |
| Cocaine/Crack Use | **.66 (.44, .85)** | **.56 (.22, .72)** | **.50 (.43, .57)** | 5.66 | 8 | .69 |
| Illicit Stimulant Use | **.55 (.34, .72)** | **.71 (.55, .81)** | **.45 (.39, .51)** | 7.92 | 8 | .44 |
| Hallucinogen Use | **.67 (.46, .82)** | **.55 (.30, .71)** | **.50 (.44, .57)** | 16.11 | 8 | .04 |
| Inhalant Use | **.66 (.39, .84)** | **.52 (.03, .71)** | **.55 (.48, .62)** | 5.37 | 8 | .72 |
| Solvent Use | *.54 (.01, .86)* | *.66 (.02, .85)* | **.52 (.40, .65)** | 4.95 | 8 | .76 |
| Dissociative Use | .49 (.01, .92) | **.73 (.09, .87)** | **.48 (.29, .63)** | 7.93 | 8 | .44 |
| Note. Bold font indicates significant parameter estimate, *p*<.001; italic font indicates significant parameter estimate, *p*<.05. | | | | | | |

| Table S3  *Test of parameter constraints for prescription-illicit configuration in the full independent pathway model* | | | |
| --- | --- | --- | --- |
| Model Comparisons (vs. 2-2-2 model) | Wald χ^2^ | df | *p* |
| Configuration on *ace* | 18.68 | 30 | .95 |
| Configuration on *ac* | 15.10 | 20 | .77 |
| Configuration on *ae* | 16.60 | 20 | .68 |
| Configuration on *ce* | 11.37 | 20 | .94 |
| Configuration on *a* | 10.55 | 10 | .39 |
| Configuration on *c* | 1.79 | 10 | .99 |
| Configuration on *e* | 6.45 | 10 | .78 |

| Table S4  *Estimates of additive genetic (a), common (c), and individual-specific (e) environmental factors in the best-fit independent pathway model* | | | | | | |
| --- | --- | --- | --- | --- | --- | --- |
|  | Standardized Coefficients | | | | | |
| Phenotype | Common Factor Loadings | | | Drug-Specific Factor Loadings | | |
|  | *a* (95% CI) | *c* (95% CI) | *e* (95% CI) | *a* (95% CI) | *c* (95% CI) | *e* (95% CI) |
| Prescription Opioid Misuse | **0.38 (0.30-0.45)** | 0.00 (fixed) | **0.64 (0.56-0.72)** | **0.62 (0.53-0.72)** | 0.00 (fixed) | 0.25 (0.00-0.60) |
| Prescription Stimulant Misuse | **0.89 (0.86-0.93)** | 0.00 (fixed) | **0.24 (0.15-0.32)** | 0.00 (fixed) | 0.00 (fixed) | **0.39 (0.34-0.43)** |
| Prescription Sedative Misuse | **0.54 (0.48-0.61)** | 0.00 (fixed) | **0.64 (0.58-0.69)** | **0.55 (0.48-0.61)** | 0.00 (fixed) | 0.04 (0.00-0.17) |
| Heroin Use | **0.80 (0.73-0.86)** | 0.00 (fixed) | **0.38 (0.28-0.49)** | 0.00 (fixed) | 0.00 (fixed) | **0.47 (0.39-0.55)** |
| Cannabis Use | **0.85 (0.80-0.90)** | 0.00 (fixed) | 0.00 (fixed) | 0.00 (fixed) | **0.44 (0.36-0.52)** | **0.29 (0.10-0.48)** |
| Cocaine/Crack Use | **0.88 (0.84-0.92)** | 0.00 (fixed) | **0.24 (0.10-0.38)** | 0.00 (fixed) | 0.00 (fixed) | **0.41 (0.37-0.45)** |
| Illicit Stimulant Use | **0.89 (0.85-0.93)** | 0.00 (fixed) | **0.19 (0.04-0.34)** | 0.00 (fixed) | **0.31 (0.22-0.40)** | **0.29 (0.18-0.39)** |
| Hallucinogen Use | **0.87 (0.84-0.90)** | 0.00 (fixed) | **0.18 (0.11-0.26)** | 0.00 (fixed) | 0.00 (fixed) | **0.46 (0.43-0.50)** |
| Inhalant Use | **0.73 (0.69-0.78)** | 0.00 (fixed) | **0.29 (0.21-0.37)** | 0.00 (fixed) | **0.48 (0.41-0.55)** | **0.38 (0.27-0.50)** |
| Solvent Use | **0.46 (0.38-0.54)** | 0.00 (fixed) | **0.33 (0.21-0.45)** | 0.00 (fixed) | **0.71 (0.62-0.81)** | **0.41 (0.23-0.59)** |
| Dissociative Use | **0.84 (0.78-0.90)** | 0.00 (fixed) | **0.34 (0.19-0.49)** | 0.00 (fixed) | 0.00 (fixed) | **0.43 (0.34-0.52)** |
|  | Variance Estimates | | | | | |
| Phenotype | Common Factor Loadings | | | Drug-Specific Factor Loadings | | |
|  | *a^2^* | *c^2^* | *e^2^* | *a^2^* | *c^2^* | *e^2^* |
| Prescription Opioid Misuse | **0.14** | 0.00 (fixed) | **0.41** | **0.39** | 0.00 (fixed) | 0.06 |
| Prescription Stimulant Misuse | **0.80** | 0.00 (fixed) | **0.06** | 0.00 (fixed) | 0.00 (fixed) | **0.15** |
| Prescription Sedative Misuse | **0.29** | 0.00 (fixed) | **0.41** | **0.30** | 0.00 (fixed) | 0.00 |
| Heroin Use | **0.64** | 0.00 (fixed) | **0.15** | 0.00 (fixed) | 0.00 (fixed) | **0.22** |
| Cannabis Use | **0.72** | 0.00 (fixed) | 0.00 (fixed) | 0.00 (fixed) | **0.19** | **0.08** |
| Cocaine/Crack Use | **0.77** | 0.00 (fixed) | **0.06** | 0.00 (fixed) | 0.00 (fixed) | **0.17** |
| Illicit Stimulant Use | **0.79** | 0.00 (fixed) | **0.04** | 0.00 (fixed) | **0.09** | **0.08** |
| Hallucinogens Use | **0.75** | 0.00 (fixed) | **0.03** | 0.00 (fixed) | 0.00 (fixed) | **0.21** |
| Inhalant Use | **0.54** | 0.00 (fixed) | **0.09** | 0.00 (fixed) | **0.23** | **0.15** |
| Solvent Use | **0.21** | 0.00 (fixed) | **0.11** | 0.00 (fixed) | **0.51** | **0.17** |
| Dissociative Use | **0.70** | 0.00 (fixed) | **0.12** | 0.00 (fixed) | 0.00 (fixed) | **0.18** |
| Note. Bold font indicates significant parameter estimate, *p*<.001; italic font indicates significant parameter estimate, *p*<.05. | | | | | | |

| Table S5  *Estimates of additive genetic (a), common (c), and individual-specific (e) environmental factors in the best-fit one-factor common pathway model* | | | | |
| --- | --- | --- | --- | --- |
|  | Standardized Coefficients | | | |
| Phenotype | Common Factor Loadings | Drug-Specific Factor Loadings* | | |
|  | General Phenotype | *a* (95% CI) | *c* (95% CI) | *e* (95% CI) |
| Prescription Opioid Misuse | **0.50 (0.44-0.55)** | **0.59 (0.47-0.71)** | 0.00 (fixed) | **0.81 (0.73-0.89)** |
| Prescription Stimulant Misuse | **0.90 (0.89-0.92)** | **0.64 (0.43-0.85)** | 0.00 (fixed) | **0.77 (0.58-0.96)** |
| Prescription Sedative Misuse | **0.67 (0.63-0.72)** | **0.63 (0.51-0.75)** | 0.00 (fixed) | **0.78 (0.68-0.87)** |
| Heroin Use | **0.89 (0.84-0.94)** | 0.00 (fixed) | *0.80 (0.25-1.00)* | *0.60 (0.20-1.00)* |
| Cannabis Use | **0.76 (0.73-0.80)** | **0.81 (0.75-0.87)** | 0.00 (fixed) | **0.59 (0.50-0.67)** |
| Cocaine/Crack Use | **0.92 (0.90-0.94)** | 0.00 (fixed) | **0.72 (0.49-0.95)** | **0.70 (0.48-0.91)** |
| Illicit Stimulant Use | **0.92 (0.90-0.94)** | 0.00 (fixed) | **0.95 (0.85-1.00)** | *0.32 (0.11-0.52)* |
| Hallucinogen Use | **0.89 (0.86-0.91)** | 0.00 (fixed) | **0.76 (0.61-0.90)** | **0.66 (0.47-0.84)** |
| Inhalant Use | **0.77 (0.73-0.80)** | **0.76 (0.65-0.87)** | 0.00 (fixed) | **0.65 (0.52-0.78)** |
| Solvent Use | **0.54 (0.46-0.61)** | 0.00 (fixed) | **0.82 (0.71-0.92)** | **0.58 (0.44-0.71)** |
| Dissociative Use | **0.91 (0.86-0.96)** | 0.00 (fixed) | *0.84 (0.28-1.00)* | *0.54 (0.10-0.97)* |
|  |  | General Phenotype Variance Components | | |
| General Phenotype | **1.00 (fixed)** | **0.68 (0.56-0.80)** | **0.58 (0.45-0.71)** | **0.45 (0.40-0.50)** |
|  | Variance Estimates | | | |
| Phenotype | Common Factor Loadings | Drug-Specific Factor Loadings | | |
|  | General Phenotype | *a^2^* | *c^2^* | *e^2^* |
| Prescription Opioid Misuse | **0.25** | **0.26** | 0.00 (fixed) | **0.50** |
| Prescription Stimulant Misuse | **0.82** | **0.07** | 0.00 (fixed) | **0.11** |
| Prescription Sedative Misuse | **0.45** | **0.22** | 0.00 (fixed) | **0.33** |
| Heroin Use | **0.80** | 0.00 (fixed) | *0.13* | *0.07* |
| Cannabis Use | **0.58** | **0.28** | 0.00 (fixed) | **0.14** |
| Cocaine/Crack Use | **0.84** | 0.00 (fixed) | **0.08** | **0.08** |
| Illicit Stimulant Use | **0.84** | 0.00 (fixed) | **0.14** | *0.02* |
| Hallucinogens Use | **0.79** | 0.00 (fixed) | **0.12** | **0.09** |
| Inhalant Use | **0.59** | **0.24** | 0.00 (fixed) | **0.17** |
| Solvent Use | **0.29** | 0.00 (fixed) | **0.48** | **0.23** |
| Dissociative Use | **0.82** | 0.00 (fixed) | *0.13* | *0.05* |
|  |  | General Phenotype Variance Components | | |
| General Phenotype | **1.00 (fixed)** | **0.47** | **0.34** | **0.19** |
| Note. Bold font indicates significant parameter estimate, *p*<.001; italic font indicates significant parameter estimate, *p*<.05; *standardized coefficients for drug-specific loadings reflect the square root of the proportion of residual variance attributable to each component. | | | | |

| Table S6  *Variance estimates of additive genetic (a^2^), common (c^2^), and individual-specific (e^2^) environmental factors in best-fit models with sex included as a covariate* | | | | | | | | | | |
| --- | --- | --- | --- | --- | --- | --- | --- | --- | --- | --- |
|  | Reduced 1-0-1 Independent Pathway Model | | | | | | | | | |
| Phenotype | Common Factor Loadings | | | | | Drug-Specific Factor Loadings | | | | |
|  | *a^2^* | | *c^2^* | | *e^2^* | *a^2^* | | *c^2^* | | *e^2^* |
| Prescription Opioid Misuse | **0.16** | | 0.00 (fixed) | | **0.43** | **0.40** | | 0.00 (fixed) | | 0.01 |
| Prescription Stimulant Misuse | **0.76** | | 0.00 (fixed) | | **0.06** | 0.00 (fixed) | | 0.00 (fixed) | | **0.15** |
| Prescription Sedative Misuse | **0.30** | | 0.00 (fixed) | | **0.39** | **0.30** | | 0.00 (fixed) | | 0.00 |
| Heroin Use | **0.61** | | 0.00 (fixed) | | **0.16** | 0.00 (fixed) | | 0.00 (fixed) | | **0.21** |
| Cannabis Use | **0.66** | | 0.00 (fixed) | | 0.00 (fixed) | 0.00 (fixed) | | **0.19** | | **0.12** |
| Cocaine/Crack Use | **0.74** | | 0.00 (fixed) | | **0.06** | 0.00 (fixed) | | 0.00 (fixed) | | **0.18** |
| Illicit Stimulant Use | **0.77** | | 0.00 (fixed) | | **0.04** | 0.00 (fixed) | | **0.09** | | **0.09** |
| Hallucinogens Use | **0.74** | | 0.00 (fixed) | | **0.04** | 0.00 (fixed) | | 0.00 (fixed) | | **0.19** |
| Inhalant Use | **0.47** | | 0.00 (fixed) | | **0.08** | 0.00 (fixed) | | **0.20** | | **0.20** |
| Solvent Use | **0.20** | | 0.00 (fixed) | | **0.12** | 0.00 (fixed) | | **0.50** | | **0.15** |
| Dissociative Use | **0.66** | | 0.00 (fixed) | | **0.13** | 0.00 (fixed) | | 0.00 (fixed) | | **0.18** |
|  | | Reduced 1-Factor Common Pathway Model | | | | | | | | |
| Phenotype | | Common Factor Loadings | | Drug-Specific Factor Loadings | | | | | | |
|  | | General Phenotype | | *a^2^* | | | *c^2^* | | *e^2^* | |
| Prescription Opioid Misuse | | **0.26** | | **0.24** | | | 0.00 (fixed) | | **.49** | |
| Prescription Stimulant Misuse | | **0.78** | | **0.08** | | | 0.00 (fixed) | | **.11** | |
| Prescription Sedative Misuse | | **0.48** | | **0.21** | | | 0.00 (fixed) | | **.31** | |
| Heroin Use | | **0.77** | | 0.00 (fixed) | | | *.13* | | .07 | |
| Cannabis Use | | **0.55** | | **.28** | | | 0.00 (fixed) | | **.14** | |
| Cocaine/Crack Use | | **0.82** | | 0.00 (fixed) | | | **.08** | | **.08** | |
| Illicit Stimulant Use | | **0.84** | | 0.00 (fixed) | | | **.14** | | .01 | |
| Hallucinogens Use | | **0.75** | | 0.00 (fixed) | | | **.12** | | **.10** | |
| Inhalant Use | | **0.55** | | **.22** | | | 0.00 (fixed) | | **.18** | |
| Solvent Use | | **0.29** | | 0.00 (fixed) | | | **.46** | | **.23** | |
| Dissociative Use | | **0.80** | | 0.00 (fixed) | | | *.12* | | ***.04*** | |
|  | |  | | General Phenotype Variance Components | | | | | | |
| General Phenotype | | **1.00 (fixed)** | | **.49** | | | **.31** | | **.21** | |
| Note. Bold font indicates significant parameter estimate, *p*<.001; italic font indicates significant parameter estimate, *p*<.05; variance estimates may not sum to 1 due to inclusion of sex as a covariate and/or rounding error. | | | | | | | | | | |
